# Detection of *N*-(1-deoxy-d-fructos-1-yl) Fumonisins B_2_ and B_3_ in Corn by High-Resolution LC-Orbitrap MS

**DOI:** 10.3390/toxins7093700

**Published:** 2015-09-16

**Authors:** Yosuke Matsuo, Kentaro Takahara, Yuki Sago, Masayo Kushiro, Hitoshi Nagashima, Hiroyuki Nakagawa

**Affiliations:** 1 National Agriculture and Food Research Organization (NARO), National Food Research Institute, 2-1-12 Kannon-dai, Tsukuba-shi, Ibaraki 305-8642, Japan; E-Mails: ymatuo@affrc.go.jp (Y.M.); sydney1219@nifty.com (Y.S.); kushirom@affrc.go.jp (M.K.); nagasima@affrc.go.jp (H.N.); 2 Thermo Fisher Scientific, C-2F, 3-9 Moriya-cho, Yokohama-shi, Kanagawa 221-0022, Japan; E-Mail: kentaro.takahara@thermofisher.com

**Keywords:** LC-Orbitrap MS, fumonisin, *Fusarium*, corn, *N*-(1-deoxy-d-fructos-1-yl) fumonisin

## Abstract

The existence of glucose conjugates of fumonisin B_2_ (FB_2_) and fumonisin B_3_ (FB_3_) in corn powder was confirmed for the first time. These “bound-fumonisins” (FB_2_ and FB_3_ bound to glucose) were identified as *N*-(1-deoxy-d-fructos-1-yl) fumonisin B_2_ (NDfrc-FB_2_) and *N*-(1-deoxy-d-fructos-1-yl) fumonisin B_3_ (NDfrc-FB_3_) respectively, based on the accurate mass measurements of characteristic ions and fragmentation patterns using high-resolution liquid chromatography-Orbitrap mass spectrometry (LC-Orbitrap MS) analysis. Treatment on NDfrc-FB_2_ and NDfrc-FB_3_ with the *o-*phthalaldehyde (OPA) reagent also supported that d-glucose binding to FB_2_ and FB_3_ molecules occurred to their primary amine residues.

## 1. Introduction

*Fusarium* fungi are known as plant pathogen infecting cereals such as wheat, barley, and corn, and some of these fungi produce mycotoxins (e.g., trichothecenes, zearalenone, and fumonisins) [1]. In Japan, *Fusarium* fungi infection is occasionally serious, as these crops are usually planted through the rainy season. Among *Fusarium* mycotoxins, fumonisins are a group of naturally-occurring mycotoxins which are typically produced by *Fusarium verticillioides* and *F. proliferatum* [2]. The most abundant fumonisin is fumonisin B_1_ (FB_1_), followed by fumonisin B_2_ (FB_2_), and fumonisin B_3_ (FB_3_). FB_1_ is a causative compound of equine leukoencephalomalacia [3] and porcine pulmonary oedema syndrome [4], and has also been confirmed to be hepatotoxic and hepatocarcinogenic in rats and mice [5,6]. Fumonisins are widely distributed geographically, and their natural occurrence in maize has been reported in various regions throughout the world [6]. A particular concern regarding fumonisins involves the higher concentrations occasionally found in maize produced and consumed by some subpopulations, such as subsistence farmers [6]. Considerable annual variations in contamination have been noted. Fumonisins also occur infrequently in other foods, including sorghum, asparagus, rice beer, and mung beans. In 2002, the FAO/WHO Joint Expert Committee on Food Additives (JECFA) established a provisional maximum tolerable daily intake (PMTDI) as 2 μg kg^−1^ bw day^−1^ for FB_1_, FB_2_, and FB_3_, either alone or in combination [6]. The European Committee concluded the establishment of a group PMTDI of 2 μg kg^−1^ bw day^−1^ for FB_1_, FB_2_, and FB_3_, combined [7].

**Figure 1:**
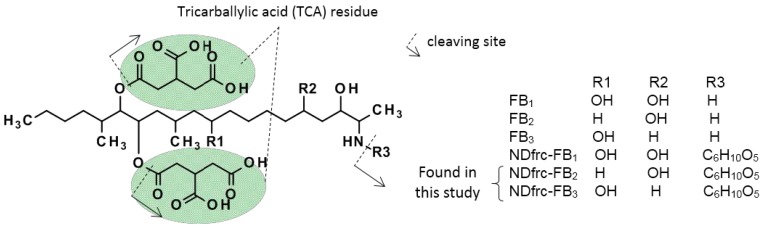
Chemical structures of FBs and their glucose conjugates.

Recently, a glucosylated derivative of deoxynivalenol (DON), DON-3-glucoside (DON3Glc) was found in cereal grain and beer [8,9], and similar compounds have also been found for several other mycotoxins [10,11]. Because these glucosylated derivatives are not detected by conventional analytical methods due to their higher polarity [12,13], they are referred to as “masked (modified) mycotoxins”. Hydrolysis of masked mycotoxins to their aglycons has also been reported [14,15], and it has been suggested that they present an additional potential risk to consumers. In the case of fumonisins, the presence of “bound-fumonisin” has been suggested by several researchers [16,17]. For instance, *N*-(1-deoxy-d-fructos-1-yl) fumonisin B_1_ (NDfrc-FB_1_) was found in corn, and was reportedly formed through a d-glucose binding reaction to the primary amine residue of the FB_1_ molecule [18]. Although proven under laboratory conditions, the NDfrc-FB_1_ occurrence at significant levels in processed samples is still controversial [19]. In addition, *in vivo* stability of this conjugate has not yet been definitively proven [17]. In order to understand the total fumonisin in foods and feeds correctly, there is a need to clarify the presence of these series of fumonisin conjugates. In this paper, authors report the existence of new glucose conjugates derived from type B fumonisins (FB_2_ and FB_3_) (Figure 1). These species were detected and identified via high resolution liquid chromatography-Orbitrap mass spectrometry (LC-Orbitrap MS), in combination with treatment using a specific reagent (*o-*phthalaldehyde, OPA).

## 2. Materials and Methods

### 2.1. Chemicals

FB_1_ and FB_2_ were purchased from Wako pure chemical Industries Ltd. (Osaka, Japan). FB_3_ was purchased from PROMEC (Tygerberg, South Africa). All other chemicals used were commercially available and of a chemically pure grade. OPA and D-glucose (>98% of chemically pure grade) were obtained from Wako. Acetonitrile (LCMS grade) was from Fisher Scientific (Waltham, MA, USA), and distilled water (LCMS grade) was obtained from Kanto Chemical (Tokyo, Japan). Ammonium acetate (chemically pure grade) was from Kanto, and acetic acid (>99.9% of chemically pure grade, not glacial) was from Wako.

### 2.2. Corn Powder Sample Contaminated with Fumonisins

Mycotoxin reference material of corn powder (batch number MTC-9999C) was purchased from Trilogy Co. Ltd (Washington, MO, USA). This material was contaminated with FB_1_, FB_2_, FB_3_, aflatoxin B_1_, aflatoxin B_2_, aflatoxin G_1_, DON, zearalenone, HT-2 toxin, and T-2 toxin. The origin of this corn was from the USA. This was a crop naturally contaminated with the above toxins. There was no inoculation of any of the toxins in this sample. The manufacturer-warranted concentrations of FB_1_, FB_2_, and FB_3_ were 19.8 ± 4.8 mg·kg^−1^ (FB_1_), 6.6 ± 2.1 mg·kg^−1^ (FB_2_), and 2.2 ± 1.6 mg·kg^−1^ (FB_3_), respectively. This material was stored at −20 °C in the dark until analysis.

### 2.3. Preparation of Stock and Working Solutions

FB_1_ and FB_2_ obtained as powder were accurately weighed on an aluminum boat on a micro scale, placed in a 10 mL brown volumetric flask, and dissolved in acetonitrile/water (1:1, *v*/*v*). FB_3_, obtained as a crystalline sample, was directly dissolved in acetonitrile/water (1:1, *v*/*v*). The concentration was normally adjusted to 100–200 mg·L^−1^, and stored in brown glass containers at 4 °C as the stock solutions. For preparation of the working solutions, each stock solution was taken in brown volumetric flasks, diluted appropriately with acetonitrile/water/acetic acid (5:94:1, *v*/*v*/*v*), and stored at 4 °C.

### 2.4. Extraction and Purification of Mycotoxins

Corn powder (8 g), 40 mL of methanol/water (75:25, *v*/*v*), and 0.4 mL of acetic acid (>99.9%) were homogenized with a POLYTRON PT3100 homogenizer (Kinematica AG., Lucerne, Switzerland) at a rate of 7000 rpm for 5 min, and centrifuged at 2000× *g* for 10 min. A portion of the supernatant (5 mL) was loaded onto a strong anion exchange column (Sep-Pak Accell Plus QMA Short Cartridge (360 mg), Waters, Milford, MA, USA) with no conditioning. Then 5 mL methanol/water (3:1, *v*/*v*) and 5 mL methanol were successively loaded on the column for washing. The FBs were eluted with 5 mL of a solution methanol/acetic acid (98:2, *v*/*v*). All of the eluent was collected in a glass tube, and the solvent was evaporated under a nitrogen gas stream at 50 °C. The residue was dissolved in 0.25 mL of acetonitrile/water/acetic acid (5:94:1, *v*/*v*/*v*) and subjected to LC-MS analysis.

### 2.5. Synthesis of N-(1-deoxy-d-fructos-1-yl) Fumonisins (NDfrc-FBs)

NDfrc-FBs was chemically prepared essentially following the procedure reported by Poling *et al.* [18]. In a glassware amber vial (2 mL volume), 0.1 mg of each fumonisin (stock solution of each was appropriately taken and evaporated), 40 mg of d-glucose, five or six beads of molecular sieve (pore size; 0.4 nm) (Millipore, Darmstadt, Germany), and 2 mL of methanol were taken, mixed, and further heated in an incubator with shaking (120 rpm) at 60 °C overnight. After the reaction, the solvent was evaporated under a nitrogen gas stream at 40 °C. The residue was re-dissolved in 2.5 mL of acetonitrile/water/acetic acid (5/94/1, *v*/*v*/*v*), and cleaned using a solid phase extraction column according to the reported procedure, with a slight modification. The re-dissolved residue (2.5 mL) was loaded on an OASIS HLB (3 cc) column (Waters), that was conditioned in advance with 3 mL of methanol and 3 mL of acetonitrile/water/acetic acid (5/94/1, *v*/*v*/*v*), successively. The column was washed with 6 mL of acetonitrile/water/acetic acid (5/94/1, *v*/*v*/*v*), and further eluted with 3 mL of methanol. This eluate was collected and evaporated to dryness under nitrogen gas at 40 °C, re-dissolved in 0.25 mL of acetonitrile/water/acetic acid (5/94/1, *v*/*v*/*v*), and subjected to LC-MS analysis.

### 2.6. LC-MS Analysis

Detection and identification of FB-glucose conjugate was conducted in accordance with author’s previous studies with the LC-Orbitrap MS instrument, “Exactive” (Thermo Fisher Scientific) [20]. LC was performed by using 0.5 mM ammonium acetate and 0.1% acetic acid aqueous solution as solvent A and 0.1% acetic acid in acetonitrile as solvent B [11]. The gradient profile used was 10% B (0–3.0 min), 90% B (18.0–22.0 min), and 10% B (22.1–29.0 min). The flow rate was set to 0.3 mL/min and the column temperature was maintained at 40 °C. The chromatographic separation was carried out on a HyPURITY C18 column (250 × 3 mm i.d., 5 μm particle size) (Thermo Fisher) with an injection volume of 0.02 mL. The Exactive mass spectrometer was operated in negative mode with a heated electrospray ionization source (HESI-II) and a spray voltage of 4.50 kV. As a typical and common fragment ion of fumonisins, ketene form of tricarballylic acid (TCA) ion [TCA−H_2_O−H]^−^ (TCAK ion [TCAK−H]^−^) was detected with higher sensitivity in negative mode than positive mode. The ion of [TCAK−H]^−^ is often selected as the primary fragment for the detection of fumonisins. The capillary and the heater temperature was 350 °C and 300 °C, respectively. The sheath gas and the auxiliary gas flow rate was adjusted as 40 and 5 (in arbitrary units), respectively. The system was operated in the range of 150–1100 *m*/*z* at a resolving power of 100,000 FWHM (full width at half maximum) (*m*/*z* 200) with an accurate mass/high resolution (AM/HR) full scan (scan event 1) and all ion fragmentation spectrum acquisition with collision energy in a single run. Fragmentation was achieved with optional CID (collision-induced dissociation) equipment, using a collision energy of 60 eV (scan event 2), that was optimized with the chemical standard of FB_1_. The external mass axis calibration without the use of specific lock mass was employed. For the mass accuracy estimation, the mass value observed as an abundant ion extracted at the apex of the chromatographic peak was used. The exact mass values (calculated and observed) of the analysts’ ions are summarized in Table 1, Table 2 and Table 3. The mass deviation is expressed either in terms of millimass units (mmu) or parts per million (ppm). The latter is calculated with the equation: ppm = 10^6^ × Δ*m*/*m*; where Δ*m* is the difference between theoretical (calculated) and observed mass, and *m* is the mass. In accordance with the European Commission guideline [21], mass deviation < 5 ppm from the calculated value was used as the criterion for compound identification. LC-Orbitrap MS is a special type of ion trap [22], and achieves a mass resolving power of up to 100,000 FWHM (*m*/*z* 200) and maintains mass accuracy (<5 ppm) even without the use of continuous internal mass correction. Therefore, it can detect and identify the various chemical compounds based on their accurate mass values calculated from the corresponding compositional formula even if those chemical standards are not available.

**Table 1:** Exact mass values of fumonisin B_1_ (FB_1_) and fumonisin B_2_(NDfrc-FB_1_) and relative fragment ions (calculated and observed) at negative polarity.

Ion	FB_1_ (RT: 15.09 min)				NDfrc-FB_1_ (RT: 14.87 min)			
	Formula	Cal. Mass (*m*/*z*) ^a^	Obs. Mass (*m*/*z*) ^b^	Error (mmu (ppm))	Formula	Cal. Mass (*m*/*z*) ^a^	Obs. Mass (*m*/*z*) ^b^	Error (mmu (ppm))
[TCA-H_2_O−H]^−^ ([TCAK−H]^−^)	C_6_H_6_O_5_	157.0142	157.0137 ^c^	−0.59 (−3.76)	C_6_H_6_O_5_	157.0142	157.0140 ^c^	−0.28 (−1.81)
			157.0135	−0.70 (−4.44)			157.0138	−0.41 (−2.59)
[M−Glc−2TCAK−H]^−^	-	-	-	-	C_22_H_47_NO_5_	404.3381	404.3378 ^c^	−0.32 (−0.78)
			-	-			404.3383	0.20 (0.50)
[M−2TCAK−H]^−^	C_22_H_47_NO_5_	404.3381	404.3379 ^c^	−0.29 (−0.71)	C_22_H_51_NO_5_	408.3695	-	-
			404.3380	−0.16 (−0.41)			-	-
[M−Glc−TCAK−H]^−^	-	-	-	-	C_28_H_53_NO_10_	562.3597	562.3607 ^c^	0.99 (1.67)
			-	-			562.3607	0.99 (1.67)
[M−TCAK−H]^−^	C_28_H_53_NO_10_	562.3597	562.3597 ^c^	0.01 (0.02)	C_34_H_63_NO_15_	724.4125	724.4136 ^c^	1.14 (1.58)
			562.3600	0.32 (0.56)			724.4137	1.20 (1.66)
[M−Glc−H]^−^	-	-	-	-	C_34_H_59_NO_15_	720.3812	720.3824 ^c^	1.19 (1.65)
			-	-			720.3823	1.13 (1.57)
[M−H]^−^	C_34_H_59_NO_15_	720.3812	720.3805 ^c^	−0.70 (−0.97)	C_40_H_69_NO_20_	882.4340	882.4349 ^c^	0.92 (1.04)
			720.3813	0.09 (0.13)			882.4362	2.20 (2.50)

^a^ Mass values calculated based on elemental formulas; ^b^ Mass values detected with the all ions fragmentation with collision energy (scan event 2); ^c^ Mass values detected by full scan (scan event 1).

### 2.7. Treatment of Fumonisins and FBs-Glucose Conjugate by OPA Reagent

For confirmation of the D-glucose binding position in the fumonisin molecule structures, treatment with the OPA reagent was performed. Since OPA reacts specifically with primary amines, this reagent is often used for the derivatization of fumonisin molecules, when they are analyzed by the conventional method with HPLC-fluorescence detection [23]. The OPA reagent was composed of 8 mg of OPA, successively dissolved in 0.2 mL of methanol, 0.01 mL of 2-mercaptoethanol, and 1 mL of 100 mM sodium tetraborate aqueous solution. The reagent was freshly prepared each week and stored in brown glass containers at 4 °C for protection from light exposure. The corn powder extract (0.05 mL) was reacted with OPA reagent (0.05 mL) by mixing, and immediately subjected to LC-MS analysis.

**Table 2:** Exact mass values of FB_2_ and NDfrc-FB_2_ and relative fragment ions (calculated and observed) at negative polarity.

Ion	FB_2_ (RT: 16.78 min)				NDfrc-FB_2_ (RT: 16.40 min)			
	Formula	Cal. Mass (*m*/*z*) ^a^	Obs. Mass (*m*/*z*) ^b^	Error (mmu (ppm))	Formula	Cal. Mass (*m*/*z*) ^a^	Obs. Mass (*m*/*z*) ^b^	Error (mmu (ppm))
[TCAK−H]^−^	C_6_H_6_O_5_	157.0142	157.0140 ^c^	−0.28 (−1.81)	C_6_H_6_O_5_	157.0142	157.0141 ^c^	−0.16 (−1.04)
			157.0138	−0.44 (−2.79)			157.0140	−0.27 (−1.72)
[M−Glc−TCAK−H]^−^	-	-	-	-	C_22_H_47_NO_4_	388.3432	388.3430 ^c^	−0.22 (−0.55)
			-	-			388.3439	0.70 (1.80)
[M−2TCAK−H]^−^	C_22_H_47_NO_4_	388.3432	-	-	C_22_H_51_NO_4_	392.3745	-	-
			388.3434 ^c^	0.18 (0.47)			-	-
[M−Glc−TCAK−H]^−^	-	-	-	-	C_28_H_53_NO_9_	546.3648	546.3652 ^c^	0.48 (0.88)
			-	-			546.3655	0.78 (1.44)
[M−TCAK−H]^−^	C_28_H_53_NO_9_	546.3648	546.3650 ^c^	0.23 (0.43)	C_34_H_63_NO_14_	708.4176	708.4188 ^c^	1.24 (1.76)
			546.3657	0.97 (1.77)			708.4183	0.70 (0.98)
[M−Glc−H]^−^	-	-	-	-	C_34_H_59_NO_14_	704.3863	704.3875 ^c^	1.17 (1.66)
			-	-			704.3879	1.60 (2.27)
[M−H]^−^	C_34_H_59_NO_14_	704.3863	704.3865 ^c^	0.20 (0.28)	C_40_H_69_NO_19_	866.4391	866.4410 ^c^	1.88 (2.17)
			704.3878	1.54 (2.18)			866.4411	2.00 (2.31)

^a^ Mass values calculated based on elemental formulas; ^b^ Mass values detected with the all ions fragmentation with collision energy (scan event 2); ^c^ Mass values detected by full scan (scan event 1).

**Table 3:** Exact mass values of FB_3_ and NDfrc-FB_3_ and relative fragment ions (calculated and observed) at negative polarity.

Ion	FB_3_ (RT: 16.09 min)				NDfrc-FB_3_ (RT: 15.68 min)			
	Formula	Cal. Mass (*m*/*z*) ^a^	Obs. Mass (m/z) ^b^	Error (mmu (ppm))	Formula	Cal. Mass (*m*/*z*) ^a^	Obs. Mass (*m*/*z*) ^b^	Error (mmu (ppm))
[TCAK−H]^−^	C_6_H_6_O_5_	157.0142	157.0139 ^c^	−0.33 (−2.11)	C_6_H_6_O_5_	157.0142	157.0142 ^c^	−0.03 (−0.16)
			157.0139	−0.38 (−2.40)			157.0143	0.02 (0.13)
[M−Glc−2TCAK−H]^−^	-	-	-	-	C_22_H_47_NO_4_	388.3432	388.3437 ^c^	0.49 (1.25)
			-	-			388.3444	1.13 (2.91)
[M−2TCAK−H]^−^	C_22_H_47_NO_4_	388.3432	-	-	C_22_H_51_NO_4_	392.3745	-	-
			388.3430 ^c^	−0.21 (−0.55)			-	-
[M−Glc−TCAK−H]^−^	-	-	-	-	C_28_H_53_NO_9_	546.3648	546.3655 ^c^	0.78 (1.44)
			-	-			546.3652	0.42 (0.77)
[M−TCAK−H]^−^	C_28_H_53_NO_9_	546.3648	546.3657 ^c^	0.97 (1.77)	C_34_H_63_NO_14_	708.4176	708.4200 ^c^	2.40 (3.39)
			546.3658	1.03 (1.88)			708.4192	1.61 (2.27)
[M−Glc−H]^−^	-	-	-	-	C_34_H_59_NO_14_	704.3863	704.3880 ^c^	1.72 (2.44)
			-	-			704.3887	2.39 (3.40)
[M−H]^−^	C_34_H_59_NO_14_	704.3863	704.3871 ^c^	0.81 (1.14)	C_40_H_69_NO_19_	866.4391	866.4417 ^c^	2.55 (2.94)
			704.3879	1.66 (2.36)			866.4420	2.91 (3.36)

^a^ Mass values calculated based on elemental formulas; ^b^ Mass values detected with the all ions fragmentation with collision energy (scan event 2); ^c^ Mass values detected by full scan (scan event 1).

## 3. Results

### 3.1. Detection of FB_1_ and NDfrc-FB_1_

**Figure 2:**
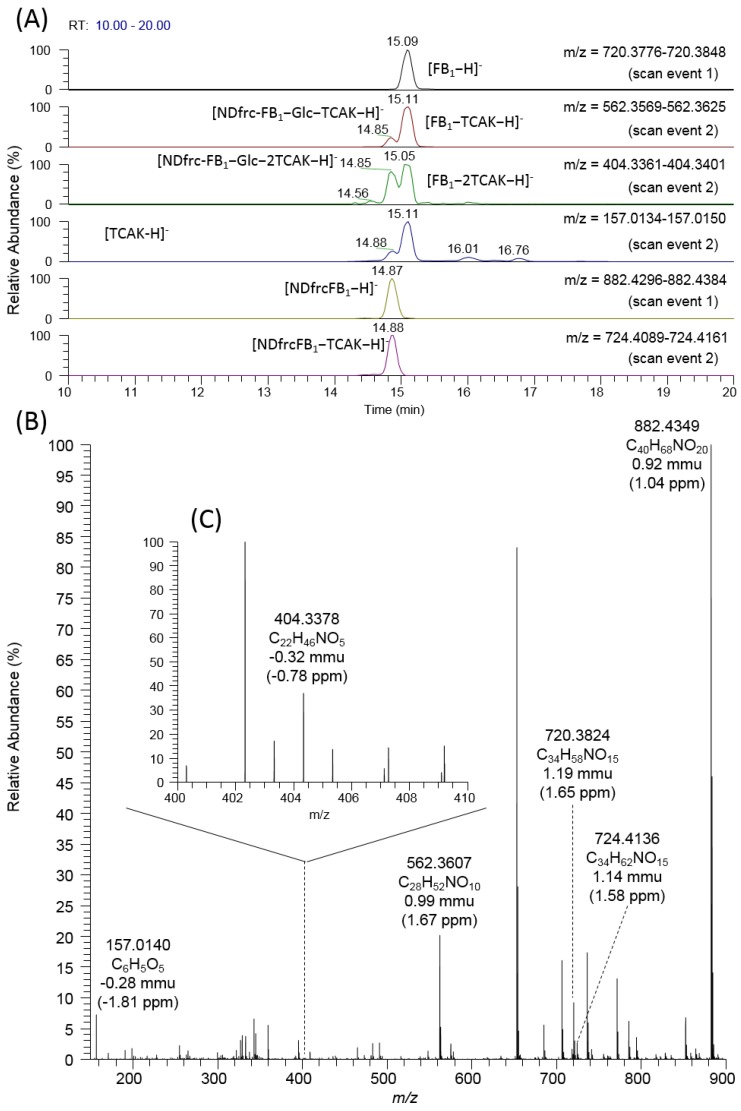
Detection and identification of NDfrc-FB_1_. Mass chromatogram with scan results (scan events 1 and 2) (**A**); full mass spectrum obtained at 14.87 min (scan event 1) (**B**); and mass range magnification of full mass spectrum (*m*/*z*: 400–410) obtained at 14.87 min (scan event 1) (**C**).

Authors first confirmed the existence of FB_1_ and NDfrc-FB_1_ in the corn powder extract, based on the full scan results using the calculated masses. In the full scan data (scan event 1), peaks corresponding to the monitor ions [FB_1_−H]^−^ (720.3812) and [NDfrc-FB_1_−H]^−^ (882.4340) were detected at 15.09 min and 14.87 min, respectively. The same peaks were observed when standard FB_1_ and authentic NDfrc-FB_1_ were injected into the LC-MS system. Regarding detection of NDfrc-FB_1_, abundant [NDfrc-FB_1_−H]^−^ (882.4349) ion was detected with a deviation of 0.92 mmu (1.04 ppm) (Figure 2B). In addition, the fragment ions [NDfrc-FB_1_−TCAK−H]^−^ (724.4136), [NDfrc-FB_1_−Glc−H]^−^ (720.3824), [NDfrc-FB_1_−Glc−TCAK−H]^−^ (562.3607), and [NDfrc-FB_1_−Glc−2TCAK−H]^−^ (404.3378) were observed, with deviations of 1.14 mmu (1.58 ppm), 1.19 mmu (1.65 ppm), 0.99 mmu (1.67 ppm) and −0.32 mmu (−0.78 ppm), respectively (Figure 2B,C). During the scan event 2, the latter two fragment ions, as well as [TCAK−H]^−^ (157.0138) provided dual peaks (at 15.11 min and 14.88 min) (Figure 2A, Table 1), indicating that similar fragmentation was occurring for FB_1_ and NDfrc-FB_1_. Although [NDfrc-FB_1_−2TCAK−H]^−^ (408.3695) was suggested as a fragment of NDfrc-FB_1_ (Table 1), a corresponding ion was not detected for NDfrc-FB_1_ (chemically synthesized or contained in corn extract). The observed mass values and their respective mass deviations from the calculated values are summarized in Table 1.

### 3.2. Detection and Identification of NDfrc-FB_2_ and NDfrc-FB_3_

**Figure 3:**
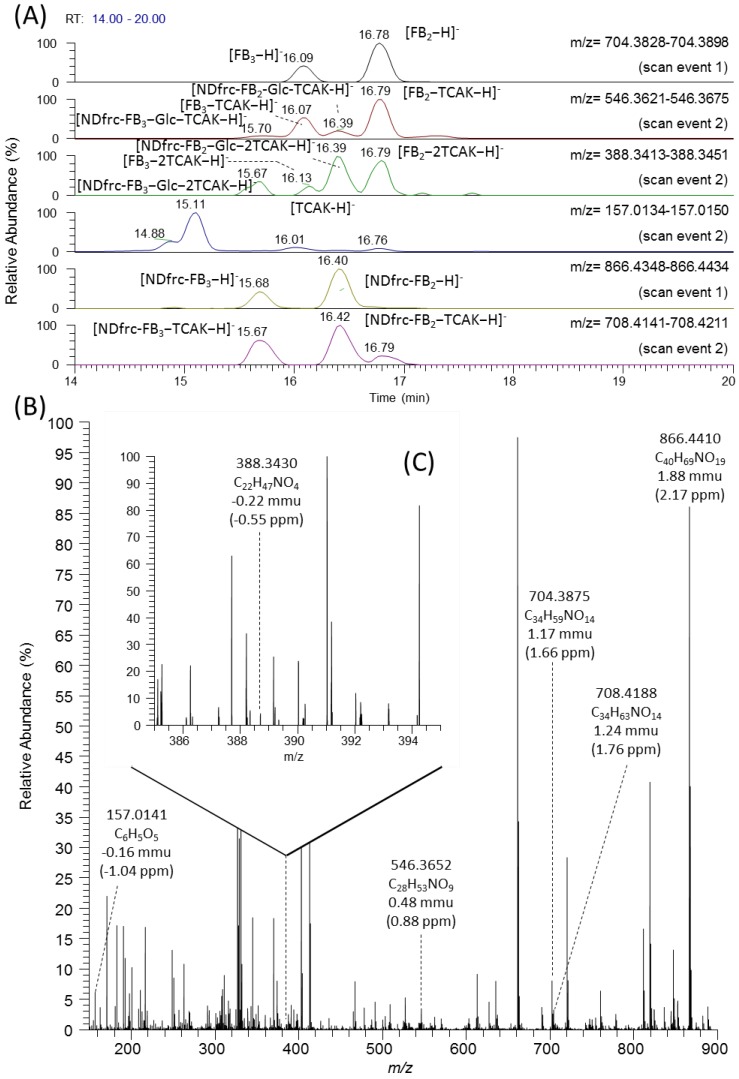
Detection and identification of NDfrc-FB_2_. Mass chromatogram with scan results (scan events 1 and 2) (**A**); full mass spectrum obtained at 16.40 min (scan event 1) (**B**); and mass range magnification of full mass spectrum (*m*/*z*: 385–395) obtained at 16.40 min (scan event 1) (**C**).

Figure 3 shows the results of screening for NDfrc-FB_2_ and NDfrc-FB_3_ in the corn powder extract. Using the same procedure as adopted for NDfrc-FB_1_, the existence of FB_2_ and FB_3_ was first confirmed based on the full scan results (scan event 1) using the calculated mass of [FB_2_−H]^−^ (704.3863). A major peak corresponding to [FB_2_−H]^−^ was detected at 16.78 min, as shown at the top of Figure 3A, and the same peak was found for the FB_2_ standard. As fragment ions of FB_2_, [FB_2_−TCAK−H]^−^, [FB_2_−2TCAK−H]^−^, and [TCAK−H]^−^ were observed (Table 2). When the full-scan results (scan event 1) were scrutinised with the calculated mass of [NDfrc-FB_2_−H]^−^ (866.4391), a major peak was detected for [NDfrc-FB_2_−H]^−^ at 16.40 min (Figure 3A), and abundant [NDfrc-FB_2_–H]^−^ (866.4410) was detected with a mass deviation of 1.88 mmu (2.17 ppm) (Figure 3B). In addition, the fragment ions [NDfrc-FB_2_–TCAK−H]^−^ (708.4188), [NDfrc-FB_2_−Glc−H]^−^ (704.3875), [NDfrc-FB_2_−Glc−TCAK−H]^−^ (546.3652), [NDfrc-FB_2_−Glc−2TCAK−H]^−^ (388.3430) were observed with deviations of 1.24 mmu (1.76 ppm), 1.17 mmu (1.66 ppm), 0.48 mmu (0.88 ppm), and −0.22 mmu (−0.55 ppm), respectively (Figure 3B, Table 2). Due to the low intensities of the signals, several fragment ions (Figure 3C) were observed only in the magnified spectra. Two peaks at 16.79 min and 16.39 min were detected for the monitor ions [NDfrc-FB_2_−Glc−TCAK−H]^−^ (546.3648) and [NDfrc-FB_2_−Glc−2TCAK−H]^−^ (388.3432) with scan event 2 (Figure 3A), suggesting that similar fragmentation was occurring for FB_2_ and NDfrc-FB_2_. During scan event 2, fragments corresponding to [NDfrc-FB_2_−TCAK−H]^−^ and [NDfrc-FB_2_−Glc−2TCAK−H]^−^ were observed (Table 2). The respective mass values of these fragments were 708.4183 mmu and 388.3439 mmu, with mass deviations from the calculated values of 0.70 mmu (0.98 ppm) and 0.70 mmu (1.80 ppm), respectively. In the case of screening for NDfrc-FB_3_, a major peak for [NDfrc-FB_3_−H]^−^ was observed at 15.68 min (scan event 1) (Figure 3A), and a fragmentation pattern similar to that of NDfrc-FB_2_ was also confirmed (details shown in Table 3). There was no difference in the fragmentation patterns of FB_2_ and FB_3_, as [FB_3_−TCAK−H]^−^, [FB_3_−2TCAK−H]^−^, and [TCAK−H]^−^ were observed as the corresponding fragment ions. Based on the data described above, authors were convinced that both NDfrc-FB_2_ and NDfrc-FB_3_ were contained in the corn powder extract.

### 3.3. Structures of NDfrc-FB_2_ and NDfrc-FB_3_

**Figure 4:**
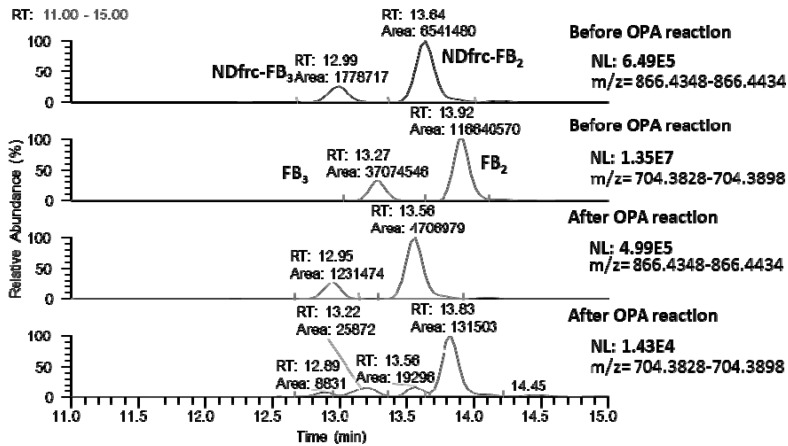
Chromatograms of NDfrc-FB_2_ (NDfrc-FB_3_) and FB_2_ (FB_3_) in corn powder extract before and after the treatment with OPA reagent (scan event 1).

Figure 4 shows the LC-MS chromatograms of the NDfrc-FB_2_ (NDfrc-FB_3_) and FB_2_ (FB_3_) detected in the corn powder extract before and after treatment with OPA reagent (scan event 1). If the structures of NDfrc-FB_2_ and NDfrc-FB_3_ were similar to that of NDfrc-FB_1_ (glucose bound to the primary amine of the fumonisin molecule), it was considered that OPA would not react with these species. As shown in Figure 4, the signal intensities of FB_2_ and FB_3_ were decreased by the OPA treatment, whereas those of NDfrc-FB_2_ and NDfrc-FB_3_ were not. The peak area ratios of NDfrc-FB_2_ (NDfrc-FB_3_), before to after the OPA reaction, were 1.44–1.46. On the other hand, these ratios of FB_2_ (FB_3_), before to after the OPA reaction were 281.4–1372.1. These results indicate that the primary amine residue was occupied by glucose conjugation in the molecules of NDfrc-FB_2_ (NDfrc-FB_3_). The slight shift observed for the fumonisin peaks was attributed to the increase in methanol concentration in the samples following the OPA treatment.

**Figure 5:**
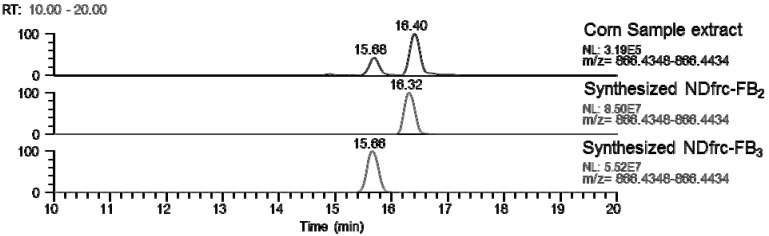
Chromatograms of NDfrc-FB_2_ and NDfrc-FB_3_ in corn powder extract in comparison with the chemically synthesized species (scan event 1).

In order to confirm the retention time and fragmentation profiles of NDfrc-FB_2_ and NDfrc-FB_3_ during the LC-Orbitrap MS analysis, these compounds were chemically synthesized with the standards of FB_2_ and FB_3_ with reference to the Poling *et al.* [18] report. Figure 5 shows the chromatograms of NDfrc-FB_2_ and NDfrc-FB_3_ in the corn powder extract and for chemically synthesized species. The mass fragmentation profiles of the synthesized NDfrc-FB_2_ (NDfrc-FB_3_) were in agreement with those of NDfrc-FB_2_ (NDfrc-FB_3_) detected in the corn sample extract (Figure S1). These results indicate that NDfrc-FB2 (and NDfrc-FB_3_) appear to be formed though a non-enzymatic reaction between FB_2_ (and FB_3_) and D-glucose. In addition, these synthesized NDfrc-FB_2_ and NDfrc-FB_3_ did not react with the OPA reagent (details not shown). Because the synthesized NDfrc-FB_2_ (and NDfrc-FB_3_) were not sufficiently pure, and contained remaining FB_2_ (and FB_3_), they could not be used for performing the quantitative analysis.

## 4. Discussion

In 2002, Poling *et al.* [18] reported that NDfrc-FB_1_ was formed through a non-enzymatic reaction between FB_1_ and glucose. Hence, authors presumed that FB_2_ and FB_3_ could also react with d-glucose non-enzymatically to form glucose conjugates such as NDfrc-FB_1_. In the current study, the existence of FB_2_-glucose and FB_3_-glucose conjugates (suggested to be NDfrc-FB_2_ and NDfrc-FB_3_) in corn powder extract was confirmed by LC-Orbitrap MS. In order to confirm that the glucose conjugation occurred at the amine residue of the FB_1_ molecule, Lu *et al.* [24] treated the FB_1_-glucose conjugate with OPA reagent. In the same manner, NDfrc-FB_2_ (and NDfrc-FB_3_) was treated with OPA in the current study. When analyzed by LC-Orbitrap MS, the peaks suggested to be NDfrc-FB_2_ and NDfrc-FB_3_ were scarcely reduced after OPA treatment, whereas those of FB_2_ and FB_3_ were reduced substantially. Based on these observations, accompanied with the high-resolution MS spectrum data described above, authors became convinced that the conjugates detected in this study should correctly correspond to NDfrc-FB_2_ and NDfrc-FB_3_. Additionally, NDfrc-FB_2_ and NDfrc-FB_3_ were observed at a different lot number of Trilogy mycotoxin reference materials (MTC-9999A and MTC-9999E) (Figure S2).

In our previous studies, several trichothecene glucosides (*O*-glucoside conjugates) were detected in a corn reference material sample of the same line from Trilogy Co. Ltd (although the batch number was different) [11,20,22]. Therefore, authors initially screened for the presence of *O*-glucoside conjugates of FB_1_–FB_3_. After treating the corn powder extract with the OPA reagent, the full MS scan data was scrutinized with the calculated mass values of C_50_H_77_NSO_21_ (FB_1_-*O*-glucoside-OPA) and C_50_H_77_NSO_20_ (FB_2_ (FB_3_)-*O*-glucoside-OPA), respectively. However, no peaks were detected. Based on these results, it appears that fumonisins are not enzymatically glucosylated, but chemically bound to glucose in-plant, which differs from the case of trichothecenes.

Among the fumonisin isomers, there are several different groups, such as fumonisin A (FA) [25] and fumonisin C (FC) [26] in addition to fumonisin B (FB_1_–FB_3_). It is suggested that FA hardly reacts with d-glucose, because its amine residue is acetylated. In contrast, FC appears to react with d-glucose non-enzymatically via the free primary amine harbored in its structure. NDfrc-FB_1_ was found from the cooked maize with heat [27], whereas NDfrc-FB_1_, NDfrc-FB_2_, and NDfrc-FB_3_ were found in the corn powder (not cooked sample) used in this study. These conjugates appeared to be formed through a non-enzymatic reaction between fumonisins (FB_2_ and FB_3_) and glucose, as reported for FB_1_ [18]. Therefore, it seems likely that high temperature (around the cooking conditions) is not indispensable for the formation of NDfrc-FBs. Once the corn grains are harvested, they are normally dried, stored at the keeping place, and ground if necessary. In the drying process, if the corn grains containing fumonisins were subjected to some heat (for promoting to eliminate the moisture), NDfrc-FB might be formed. From the standpoint of toxicity, NDfrc-FB_1_ has been reported as less toxic, compared to FB_1_ [28]. The toxicity of NDfrc-FB_2_ and NDfrc-FB_3_ is suggested to be lower than FB_2_ and FB_3_ with reference to NDfrc-FB_1_. It was also reported that NDfrc-FB_1_ was partly converted back to FB_1_ in the gastrointestinal tract of rats [29]. On the other hand, Cirlini *et al.* [30] reported that NDfrc-FB_1_ was not reduced to FB_1_ *in vitro* digestion model [30]. As one possible factor reducing NDfrc-FB_1_ to FB_1_, the involvement of rat microbiota is inferred. Although crucial microbes reducing NDfrc-FB_1_ are unknown, the microbiota in the gastrointestinal tract is greatly different amongst host species, area, and age [31,32]. Another important question concerns the amount of these fumonisin conjugates that are present. However, in the current study, it was not possible to estimate the amounts of these compounds due to a lack in the pure (purified) chemical standards.

## 5. Conclusions

In conclusion, new glucose conjugates of FB_2_ and FB_3_ (NDfrc-FB_2_ and NDfrc-FB_3_) were detected for the first time in the corn sample in this study. These conjugates appeared to be formed through a non-enzymatic reaction between fumonisins (FB_2_ and FB_3_) and glucose. Although these reactions are similar to Maillard reaction, NDfrc-FBs are not reduced to FBs in contrast to the conjugate reaction of amino acid and D-glucose [33] even if they are treated with alkali. Considering that NDfrc-FB seems to be less toxic than FB, some food processing procedures (for example, promotion of Maillard reaction) can be suggested to mitigate the fumonisin toxicity, as examined previously [19]. The existence of acyl-fumonisin B_1_ [17] and fumonisins bound to starch (hidden fumonisins) [34] has also been reported by other researchers. Although the use of the hydrolyzed FBs (HFBs) obtained with either alkaline or enzymatic treatment has been proposed for quantitation of the total fumonisins in starch [17], NDfrc-FBs should not be determined with these methods. Since the PMTDI value was designated as 2 μg·kg^−1^ bw·day^−1^ in the JECFA [6], the existence of those fumonisin conjugates may be taken into account for the establishment of this value. In order to estimate the potential risk of fumonisins, further studies on the prevalence of these and other conjugates in foods, as well as their relevance for human health is needed.

## Supplementary Materials

Supplementary materials can be accessed at: http://www.mdpi.com/2072-6651/7/9/3700/s1.
