# Supplementary material for: Detection of N-(1-deoxy-d-fructos-1-yl) Fumonisins B2 and B3 in Corn by High-Resolution LC-Orbitrap MS
Source: Toxins (Basel). 2015 Sep 16;7(9):3700–14. doi: 10.3390/toxins7093700 (PMC4591641; doi:10.3390/toxins7093700)
Supplement: Supplementary file 1 [file toxins-07-03700-s001.pdf]

Supplementary Materials

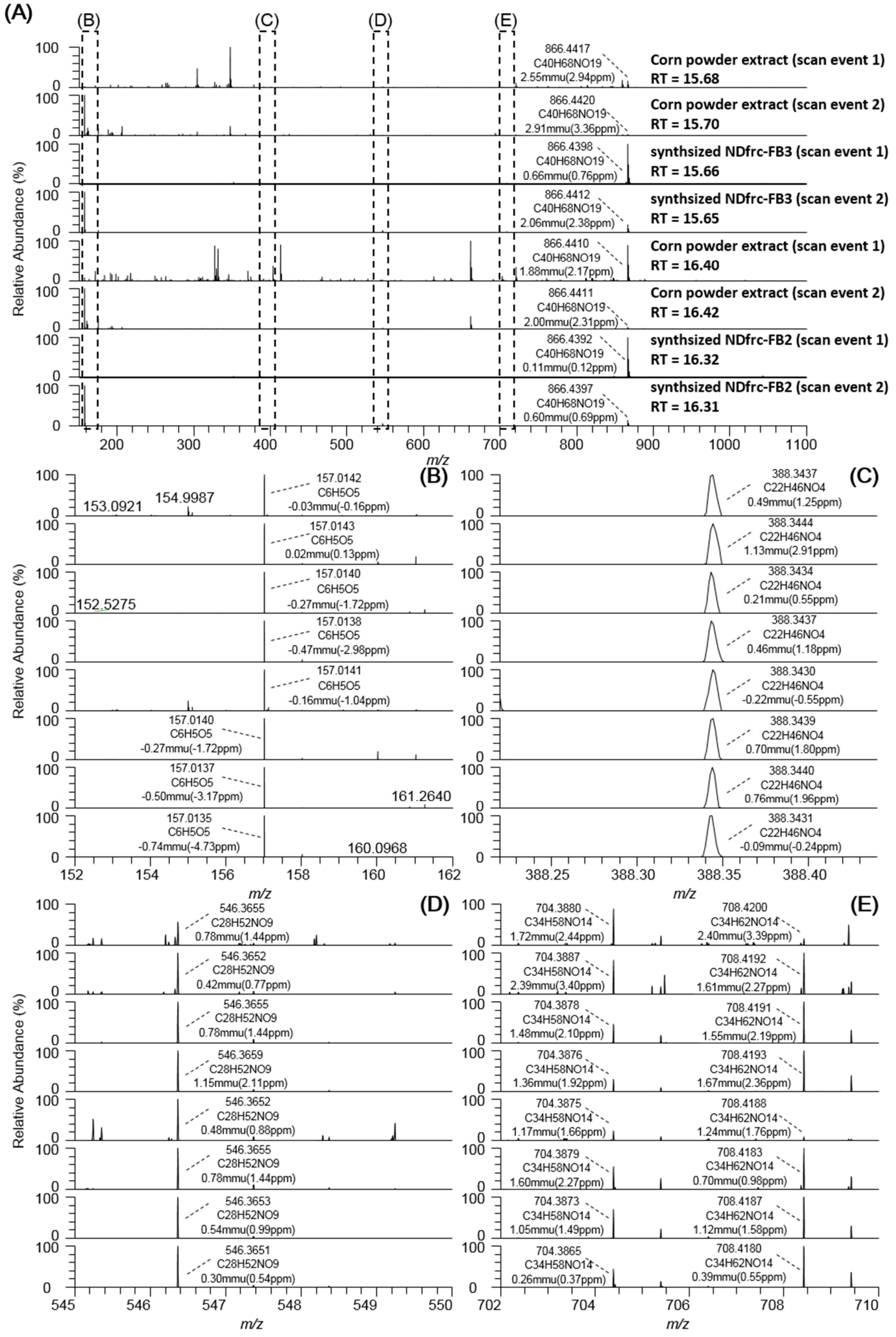

Figure S1. Precursor and product ion spectra of NDfrc-FB_2 and NDfrc-FB_3 (in corn powder extract in comparison with those chemically synthesized), and characteristic peak assignment of NDfrc-FB_2 and NDfrc-FB_3. (**A**) full mass range spectra (**B**–**E**) magnification of NDfrc-FBs fragments' spectra.

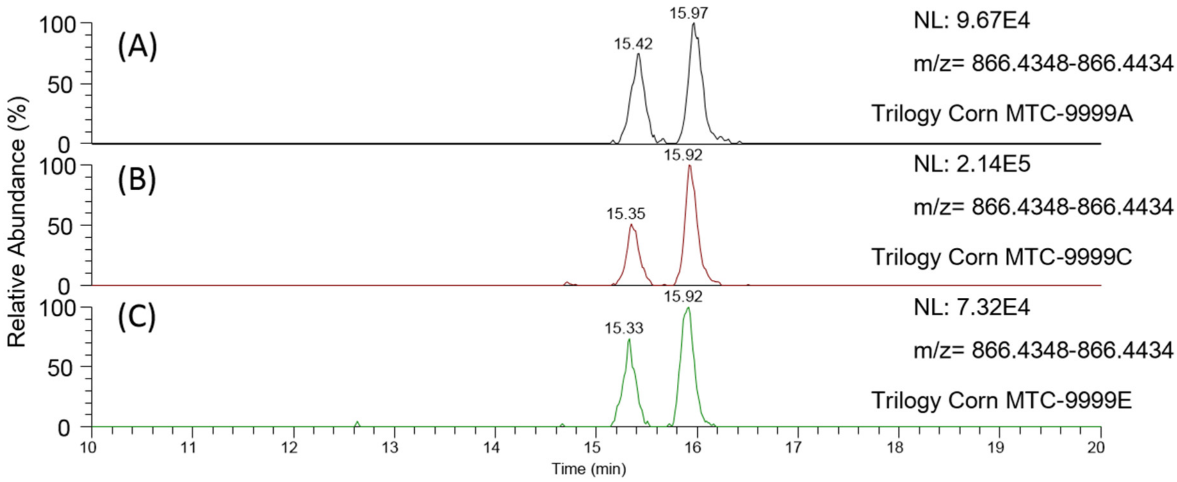

Figure S2. Chromatograms of NDfrc-FB_2 (NDfrc-FB_3) and FB_2 (FB_3) in Trilogy corn powder extracts (batch number MTC-9999A (**A**); batch number MTC-9999C (**B**) and batch number MTC-9999E (**C**)).
